# Synergistic Removal of Static and Dynamic *Staphylococcus aureus* Biofilms by Combined Treatment with a Bacteriophage Endolysin and a Polysaccharide Depolymerase

**DOI:** 10.3390/v10080438

**Published:** 2018-08-18

**Authors:** Nanna M. C. Olsen, Elowine Thiran, Tobias Hasler, Thomas Vanzieleghem, Georgios N. Belibasakis, Jacques Mahillon, Martin J. Loessner, Mathias Schmelcher

**Affiliations:** 1 Institute of Food, Nutrition and Health, ETH Zurich, Schmelzbergstrasse 7, 8092 Zurich, Switzerland; nanna.olsen@bio.ku.dk (N.M.C.O.); elowine.thiran@gmail.com (E.T.); tobias.hasler@uzh.ch (T.H.); martin.loessner@ethz.ch (M.J.L.); 2 Section of Microbiology, Department of Biology, University of Copenhagen, Universitetsparken 15, 2100 Copenhagen Ø, Denmark; 3 Earth and Life Institute, Université Catholique de Louvain, Croix du Sud 2/L7.05.12, 1348 Louvain-la-Neuve, Belgium; t.vanzieleghem@onelife-bf.com (T.V.); jacques.mahillon@uclouvain.be (J.M.); 4 Division of Oral Diseases, Department of Dental Medicine, Karolinska Institutet, Alfred Nobels Allé 8, 141 04 Huddinge, Sweden; george.belibasakis@ki.se; 5 Institute of Oral Biology, Center of Dental Medicine, University of Zurich, Plattenstrasse 11, 8032 Zurich, Switzerland

**Keywords:** endolysin, depolymerase, biofilm, *S. aureus*, synergy, antimicrobial, dynamic model, flow cell

## Abstract

*Staphylococcus aureus* is an important pathogen and biofilm former. Biofilms cause problems in clinics and food production and are highly recalcitrant to antibiotics and sanitizers. Bacteriophage endolysins kill bacteria by degrading their cell wall and are therefore deemed promising antimicrobials and anti-biofilm agents. Depolymerases targeting polysaccharides in the extracellular matrix have been suggested as parts of a multi-enzyme approach to eradicate biofilms. The efficacy of endolysins and depolymerases against *S. aureus* biofilms in static models has been demonstrated. However, there is a lack of studies evaluating their activity against biofilms grown under more realistic conditions. Here, we investigated the efficacy of the endolysin LysK and the poly-*N*-acetylglucosamine depolymerase DA7 against staphylococcal biofilms in static and dynamic (flow cell-based) models. LysK showed activity against multiple *S. aureus* strains, and both LysK and DA7 removed static and dynamic biofilms from polystyrene and glass surfaces at low micromolar and nanomolar concentrations, respectively. When combined, the enzymes acted synergistically, as demonstrated by crystal violet staining of static biofilms, significantly reducing viable cell counts compared to individual enzyme treatment in the dynamic model, and confocal laser scanning microscopy. Overall, our results suggest that LysK and DA7 are potent anti-biofilm agents, alone and in combination.

## 1. Introduction

*Staphylococcus aureus* is an opportunistic bacterial pathogen which can cause a broad variety of infectious diseases in both humans and animals. The long list of medical conditions caused by these Gram-positive bacteria includes abscesses of skin, muscles and various organs, infective endocarditis, osteomyelitis, pneumonia, and toxic shock syndrome [1,2]. *S. aureus* can also exist in polymicrobial biofilms of the oral cavity [3] and has frequently been implicated in oral infections, such as peri-implantitis [4,5]. In addition, *S. aureus* is frequently responsible for food poisoning via production of heat-stable enterotoxins [6]. Besides their important role as human pathogens, staphylococci (and *S. aureus* in particular) are a major cause of bovine mastitis, an infection of the mammary gland in cows. This leads to severe losses in milk production and quality and increased costs due to veterinary treatment and culling of animals, making it the most costly disease for the dairy industry [7]. In both human and veterinary clinics, the increasing prevalence of antibiotic-resistant strains such as methicillin-resistant *S. aureus* (MRSA) as well as the ability of staphylococci to form biofilms result in prolonged therapies and increased treatment costs [8,9].

Biofilms are sessile communities of bacteria embedded in a self-produced extracellular matrix that can grow attached to biotic and abiotic surfaces and as free-floating pellicle structures. The extracellular biofilm matrix consists of multiple components, including extracellular DNA, proteins, environmental components and exopolysaccharides, which provide structure and resilience and often constitute the major part of the matrix [10,11]. One exopolysaccharide frequently found in staphylococcal biofilms is poly-*N*-acetylglucosamine (PNAG), also referred to as polysaccharide intercellular adhesin (PIA), which is produced by enzymes encoded within the *icaADBC* operon [9]. The ability to form biofilms represents an important virulence factor for many pathogenic bacteria, including staphylococci, and is associated with an increased tolerance to antimicrobial agents and host defenses [12]. In addition, in the food industry, biofilms on food processing surfaces cause major problems. Due to their high levels of recalcitrance towards sanitizers and cleaning processes, they constitute permanent sources of contamination [13]. In *S. aureus*-induced bovine mastitis, the formation of biofilms is believed to contribute to bacterial persistence in the udder, the recurrence of intramammary infections, and the difficulty to treat these infections [14].

Endolysins are bacteriophage-encoded peptidoglycan hydrolases with the function to lyse the bacterial host cell by degrading its cell wall at the end of the lytic multiplication cycle of the phage. In their natural context, endolysins gain access to their peptidoglycan substrate from within the bacterial cell by help of cytoplasmic membrane-perforating holin proteins [15]. However, in the absence of an outer membrane in Gram-positive bacteria, these enzymes can also access the peptidoglycan and destroy the bacterial target cell from the outside, which renders them promising antibacterial agents [16,17,18]. Their major advantages as antimicrobials include their rapid killing activity against antibiotic-sensitive and -resistant strains, their specificity for their target bacteria, and their low chance of resistance development. Numerous in vitro studies and animal models of bacterial infection have demonstrated their efficacy as antimicrobial agents against multiple pathogens [19,20]. A recent study compared the efficacies of nine unique peptidoglycan hydrolases including eight phage endolysins and one bacteriocin against *S. aureus*, using multiple in vitro activity assays, a mouse model of systemic MRSA infection, and a static biofilm reduction assay [21]. The most potent endolysin within this set of enzymes, LysK [22], demonstrated high staphylolytic activity against all tested strains (including MRSA strains and other clinical isolates), protected 100% of MRSA-infected mice from death, and was the most effective endolysin at degrading *S. aureus* biofilms in the 96-well plate-based model [21].

Similar static biofilm models have also been employed to investigate the ability of other endolysins [21,23,24,25] as well as various enzymes attacking the extracellular matrix to degrade staphylococcal biofilms. The latter include DNase I, which can degrade extracellular DNA [26], the *Aggregatibacter actinomycetemcomitans*-derived PNAG depolymerase dispersin B [27,28], and the phage-derived exopolysaccharide depolymerase Dpo7 [29]. The combined application of such enzymes that simultaneously target multiple components of a biofilm, thereby potentially capitalizing on synergistic effects, has been suggested as a promising approach to prevent biofilm formation and to eradicate mature biofilms [10]. While the static biofilm models used in the aforementioned studies may provide first insights into the anti-biofilm activities of such agents, they insufficiently mimic natural conditions encountered in the human body or the food industry, where growing biofilms are often exposed to shear forces under the continuous flow of liquids. Therefore, including more sophisticated dynamic models such as flow-cell systems is desirable in studies evaluating the efficacy of anti-biofilm agents [23,30,31].

Here, we further investigate the activity of the endolysin LysK against *S. aureus* biofilms, employing both conventional static and flow-cell-based dynamic models. Moreover, we evaluate the anti-biofilm efficacy of enzymes targeting the extracellular matrix, including a PNAG depolymerase with homology to dispersin B, alone and in combination with LysK.

## 2. Materials and Methods

### 2.1. Bacterial Strains, Plasmids, and Culture Conditions

*Escherichia coli* strains XL1-Blue MRF’ and BL21-Gold(DE3) (Agilent Technologies, Santa Clara, CA, USA) were used for cloning of *A. actinomycetemcomitans*-derived genes encoding different depolymerase enzymes and expression of recombinant proteins. BL21-Gold(DE3) harboring a plasmid construct coding for the *S. aureus* phage endolysin LysK in a pET-21a backbone (EMD Biosciences, San Diego, CA, USA) served as expression strain for recombinant production of a C-terminally 6× His-tagged version of LysK [21]. *E. coli* strains were cultured at 37 °C in Luria-Bertani (LB) medium supplemented with ampicillin (100 µg/mL; for BL21-Gold(DE3)) or ampicillin and tetracycline (30 µg/mL; for XL1-Blue MRF’) for plasmid selection. *S. aureus* and *A. actinomycetemcomitans* strains used in this work are listed in Table 1. *S. aureus* was routinely cultured aerobically in tryptic soy broth (TSB; Biolife, Milan, Italy) at 37 °C, and *A. actinomycetemcomitans* was grown on Difco^TM^ Columbia Blood Agar plates (BD, Allschwil, Switzerland) at 37 °C in the presence of 10% CO_2_.

### 2.2. DNA Techniques and Cloning Procedures

Standard molecular biology techniques [36] were used for cloning of recombinant *A. actinomycetemcomitans*-derived depolymerase constructs. Colony material from 5 different *A. actinomycetemcomitans* strains (Table 1) served as templates for PCR amplification of respective genes using Phusion^®^ High-Fidelity DNA Polymerase (New England Biolabs, Allschwil, Switzerland). Primers were designed based on the nucleotide sequence of the previously described *A. actinomycetemcomitans*-derived DspB depolymerase [27]. Primer pair DA_BamHI_F (TGCAGGATCCAATTGTTGCGTAAAAGGC) and DA_SalI_R (ACTTGTCGACTTACTCATCCCC ATTCGTC) was used for generation of N-terminally 6× His-tagged constructs, and primer pair DA_NdeI_F (AGTCTGTTCACATATGAATTGTTGCGTAAAAGGC) and DA_XhoI_R (CTGATACT CGAGCTCATCCCCATTCGTC) for generation of C-terminally 6× His-tagged proteins. Resulting fragments were inserted into *Bam*HI and *Sal*I or *Nde*I and *Xho*I restriction sites of the plasmids pQE-30 (Qiagen, Hilden, Germany) or pET-21a (EMD Biosciences, San Diego, CA, USA), encoding N- or C-terminal His-tags, respectively. The plasmid constructs were introduced into *E. coli* XL1-Blue MRF’ (for pQE-30-based constructs) or *E. coli* BL21-Gold(DE3) (for pET-21a-based constructs). All sequences were verified by nucleotide sequencing (GATC, Konstanz, Germany).

### 2.3. Protein Expression and Purification

Expression of 6× His-tagged proteins in *E. coli* and purification via immobilized metal ion affinity chromatography was performed essentially as previously described [37]. In brief, bacterial cultures were grown in LB medium optimized for protein expression (LB-PE) [38] supplemented with antibiotics for plasmid selection at 37 °C. Once an OD_600nm_ of 0.5 was reached, cultures were cooled down on ice, and protein expression was induced by addition of 0.5 mM IPTG. Following incubation at 19 °C for 18 h, bacterial cells were harvested by centrifugation and resuspended in buffer A (50 mM NaH_2_PO_4_, 500 mM NaCl, 5 mM imidazole, 0.1% Tween 20, pH 8; for depolymerases) or lysis buffer (50 mM NaH_2_PO_4_, 300 mM NaCl, 10 mM imidazole, 30% glycerol, pH 8; for LysK). Cells were disrupted using a Stansted pressure cell homogenizer (SPCH-10-230V; Stansted Fluid Power, Harlow, UK). Lysates were cleared by centrifugation, and target proteins purified from the crude extracts by immobilized metal ion affinity chromatography using low-density nickel resin (ABT, Madrid, Spain) in Econo-Pac gravity flow columns (Bio-Rad, Cressier, Switzerland). Proteins were eluted with buffer B (50 mM NaH_2_PO_4_, 500 mM NaCl, 250 mM imidazole, 0.1% Tween 20, pH 8; for depolymerases) or elution buffer (50 mM NaH_2_PO_4_, 300 mM NaCl, 250 mM imidazole, 30% glycerol, pH 8; for LysK). Eluted fractions with high protein concentration were pooled, dialyzed against dialysis buffer (50 mM NaH_2_PO_4_, 100 mM NaCl, 0.005% Tween 20, pH 8 for depolymerases; 50 mM NaH_2_PO_4_, 300 mM NaCl, 30% glycerol, pH 8 for LysK) and filter-sterilized (0.2 µM). Protein identity and purity were assessed by sodium dodecyl sulfate polyacrylamide gel electrophoresis (SDS-PAGE), protein concentrations were measured spectrophotometrically (NanoDrop ND-100, NanoDrop Technologies, Wilmington, DE, USA), and protein preparations were stored on ice. 

### 2.4. Static Biofilm Model

Static biofilm reduction assays were conducted as previously described [21] to test the efficacy of LysK, *A. actinomycetemcomitans*-derived polysaccharide depolymerases, and DNase I (Sigma-Aldrich, Buchs, Switzerland) against *S. aureus* biofilms. In brief, biofilms were grown in TSB supplemented with 0.25% d(+)-glucose (TSBG) in a polystyrene 96-well plate for 24 h at 30 °C. The biofilms were washed with phosphate-buffered saline (PBS) and treated with serial dilutions of LysK for 2.5 h at 37 °C (standard protocol). In the case of the depolymerases and DNase I, the standard protocol was modified and treatment was done for 30 min at 30 °C and for 1 h at 37 °C, respectively. For synergy experiments with LysK and the depolymerase DA7, biofilms were treated for 1 h at 37 °C, representing a compromise between the two conditions used for the individual enzyme treatments. After treatment, residual biofilms were washed with PBS, stained with 0.4% crystal violet (CV), and washed again. The CV stain was dissolved in 96% ethanol, and the absorption at 595 nm (A_595nm_) of the resulting solutions was measured spectrophotometrically.

### 2.5. Dynamic Biofilm Model

For studying the efficacy of LysK and DA7 to eradicate dynamically grown *S. aureus* biofilms, a Biostream flow cell system [31,39] was employed. The flow cell consists of a molded silicone block forming a flow chamber, which is sealed on the bottom side with a glass slide (Gerhard Menzel GmbH, Braunschweig, Germany) serving as the surface for biofilm growth. Glass slides were pretreated with a mix (2:1) of 98% sulfuric acid and 30% hydrogen peroxide to remove organic and inorganic material and thoroughly washed with deionized water. The entire assembly, consisting of the flow cell, silicon tubes (1.6 mm inner diameter) connected by straight and Y-shaped Kynar connectors (Fisher Scientific, Reinach, Switzerland), and Schott Duran glass bottles equipped with two- and three-port screw caps and pressure equalization sets (VWR, Dietikon, Switzerland) was autoclaved before use. To ensure a continuous flow of medium during biofilm growth, the assembly was connected to a Minipuls Evolution peristaltic pump (Gilson, Middleton, WI, USA). To initiate biofilm growth, 20 mL of a *S. aureus* SA113 overnight culture were centrifuged (7000× *g* for 20 min), and the pellet was resuspended in PBS to an OD_600nm_ of 1. The flow cells were first filled with PBS, followed by the *S. aureus* suspension for 20 min at a flow rate of 570 μL/min (resulting in shear forces of 10^−2^ Pa), and then incubated for 20 h at 4 °C, 10 °C, 19 °C (chosen as the standard procedure after preliminary experiments), 25 °C or 37 °C under constant flow (570 μL/min) of TSBG. Flow cells were then disassembled, the glass slides containing the biofilms washed in PBS and subsequently submerged in solutions of LysK and/or DA7 or buffer as a control for 2 or 5 h at 25 °C. Following treatment, the slides were washed with PBS, and residual biofilm on the slides was quantified by CV staining as described above for the static biofilm model. Alternatively, the number of residual viable *S. aureus* cells on the slides was determined by vortexing the glass slides with 5 mL of Marienfeld Superior soda lime glass beads (Fisher Scientific) in 30 mL PBS for 30 s and plating serial dilutions of the supernatant on agar plates for enumeration of colonies.

### 2.6. Determination of Synergy between LysK and DA7

To determine synergistic effects between LysK and DA7 against *S. aureus* biofilms in a static model, 2-fold serial dilutions of both enzymes starting with their respective minimum biofilm eradication concentrations (MBECs) as highest concentrations were prepared. The MBEC was defined as the lowest concentration of an agent required to remove all visible biofilm from the polystyrene surface in the static biofilm reduction assay as described above. The efficacies of these serially diluted individual enzymes (MBECs x and y) at removing static *S. aureus* biofilms were then compared to those of 2-fold serially diluted mixtures of both enzymes at different ratios (½ x + ½ y, ¾ x + ¼ y, and ¼ x + ¾ y). For each mixture, the sum of fractional biofilm eradication concentrations (ΣFBEC; corresponding to the sum of fractional inhibitory concentrations, ΣFIC, in a classical microdilution broth synergy assay [40,41]) as a measure of synergy was calculated from the difference between the row containing the first cleared well for the enzyme mixture and the row containing the first cleared wells for the single enzymes [37]. In analogy to the classical synergy assays, enzyme combinations with a ΣFBEC below 0.5 were considered synergistic [41]. To determine synergistic effects in the dynamic model, the *S. aureus* biofilm was grown in 4 parallel flow cells as described above and treated with buffer (control), DA7 (concentration x), LysK (concentration y), or a mixture of DA7 and LysK (¼ x + ¾ y) for 2 h at room temperature. For each treatment, residual viable *S. aureus* cells were determined as described above.

### 2.7. Confocal Laser Scanning Microscopy

For confocal laser scanning microscopy (CLSM), *S. aureus* biofilms were grown essentially as described above for the dynamic biofilm model with some modifications. Instead of Biostream flow cells, 8-cell µ-Slide VI^0.4^ flow cells (IBIDI, Martinsried, Germany) were used. Due to the smaller dimensions of these cells, the flow rate was reduced to 60 μL/min in an effort to achieve similar shear forces (10^−2^ Pa) as in the Biostream setup. Biofilms were washed with PBS at the same flow rate before filling the cells with enzyme solution and incubating them without flow for 2 h at 25 °C. After treatment, the cells were washed again, and residual biofilms in the cells were stained with live/dead stain (LIVE/DEAD BacLight; Life Technologies, Carlsbad, CA, USA) diluted 1:1 with PBS for 15 min. After another wash with PBS, biofilms were visualized by CLSM, using a Leica TCS SPE system (Leica Microsystems, Wetzlar, Germany). Three-dimensional reconstructions of biofilms were generated in silico from recorded z-stacks using the software ImageJ [42].

### 2.8. Statistical Analysis

One-way analysis of variance (ANOVA) with a post hoc Tukey honestly significant difference (HSD) test was used for multiple means comparisons or Welch’s *t*-test for two-mean comparisons in dynamic biofilm reduction experiments. 

## 3. Results

### 3.1. The Bacteriophage Endolysin LysK Is Active against Biofilms of Multiple S. aureus Strains

**Figure 1:**
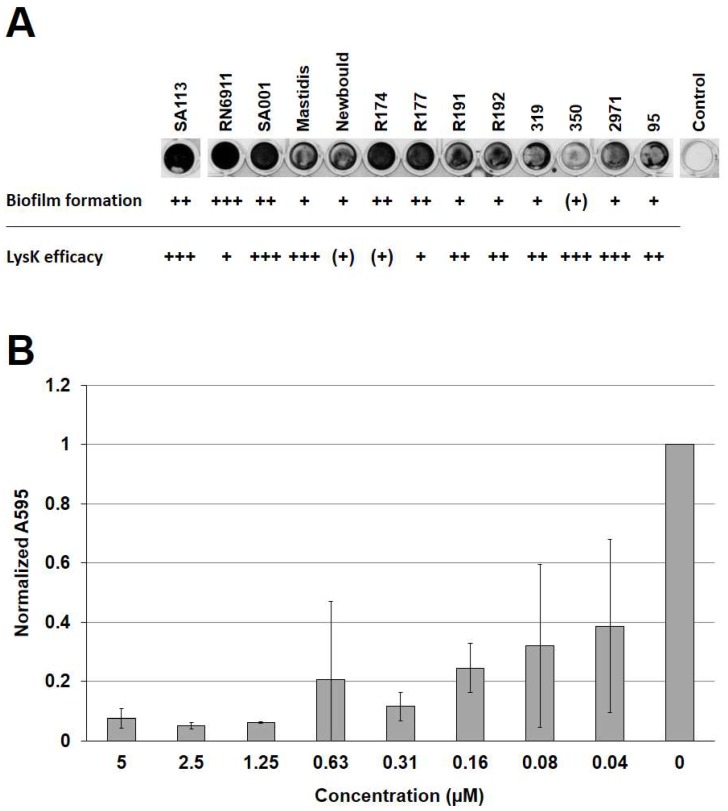
Activity of LysK against biofilms of various *S. aureus* strains in a static model. (**A**) Biofilm formation and susceptibility to LysK treatment of various *S. aureus* strains. Biofilms were grown statically in 96-well plates for 24 h at 30 °C and stained with CV. The strains were rated for their biofilm forming ability based on A_595nm_ measurements of solubilized CV as follows: +++, very strong (A_595nm_ > 3); ++, strong (A_595nm_ > 1); +, moderate (A_595nm_ > 0.2); (+), weak (A_595nm_ ≤ 0.2). At least three experiments were conducted, and one representative well for each strain is shown (**top**). Efficacy of LysK against biofilms of each strain was determined as exemplified for SA113 in (**B**) and rated as follows (**bottom**): +++, > 40% reduction in A_595nm_ compared to the control at a concentration (c) ≤ 0.08 µM; ++, > 40% reduction at c ≤ 0.31 µM; +, > 40% reduction at c ≤ 1.25 µM; (+), no reduction > 40% even at c > 1.25 µM. (**B**) *S. aureus* SA113 biofilms grown in a 96-well plate for 24 h at 30 °C were treated with LysK at different concentrations or buffer as a control (0) for 2.5 h, and residual biofilms after treatment were stained with crystal violet (CV). After dissolving the CV in 96% ethanol, the A_595nm_ of each well was measured spectrophotometrically. All values were normalized to the control. Error bars indicate standard deviations from three independent experiments.

To determine the activity of LysK against biofilms of multiple *S. aureus* strains, a selection of strains from our laboratory collection (Table 1) was first assessed for their ability to form biofilms on polystyrene surfaces in a static 96-well plate-based model. These included SA113, a reportedly strong biofilm former and producer of PNAG [32,43], which served as a positive control in this experiment; three bovine mastitis isolates from different geographic regions; multiple food isolates; and RN6911, a mutant strain deficient of the accessory gene regulator (*agr*) [33]. Expression of this regulator results in enhanced biofilm dispersal [44] and, therefore, the mutant strain was expected to be a strong biofilm producer. In fact, RN6911 demonstrated the best biofilm forming ability of all tested strains, as revealed by CV staining of biofilms grown for 24 h at 30 °C (Figure 1A). In addition, SA113, the mastitis strain SA001, and the food isolates R174 and R177 were found to produce strong biofilms. The remaining strains were classified as moderate biofilm formers, with the exception of strain 350, which only produced weak biofilms. We then tested the efficacy of LysK against biofilms of each of these strains in the static model. To this end, the biofilms were exposed to two-fold serial dilutions of the endolysin for 2.5 h, and residual biofilms were stained with CV and quantified spectrophotometrically, as exemplified for SA113 in Figure 1B. The susceptibility of each strain to LysK degradation was rated based on the concentration-dependent reduction in biofilm mass (Figure 1A). The majority of strains were highly susceptible to the action of the endolysin, with concentrations as low as 40 nM causing a reduction in biofilm mass. Only strains Newbould and R174 showed a higher degree of recalcitrance to the enzyme, with none of the tested enzyme concentrations reducing the biofilm by more than 40%.

### 3.2. LysK Degrades S. aureus Biofilms Grown under Dynamic Conditions

**Figure 2:**
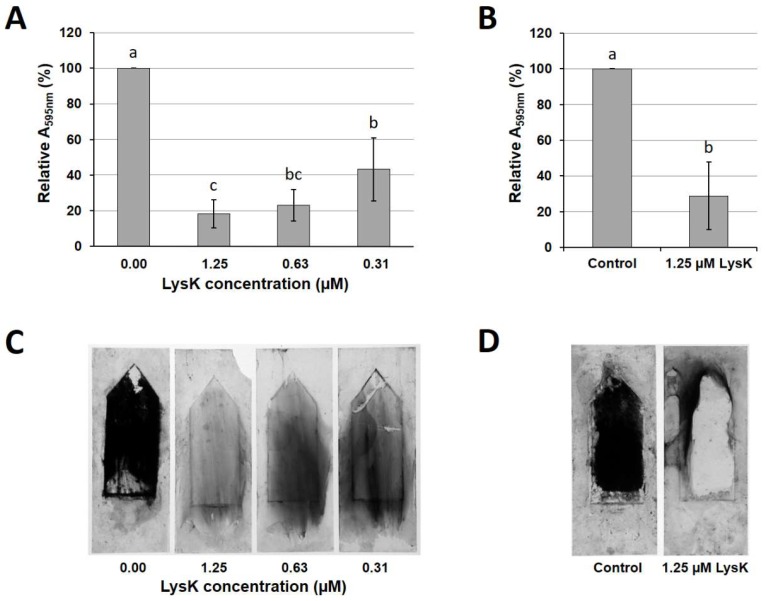
Efficacy of LysK against dynamic *S. aureus* SA113 biofilms grown on glass surfaces in the Biostream flow cell. Biofilms were grown for 20 h at 19 °C under continuous flow of medium. Glass slides were then submerged in solutions of LysK at different concentrations or buffer as a control for 5 h (**A**,**C**) or 2 h (**B**,**D**), and stained with crystal violet. The stain was dissolved in 96% ethanol and the A_595nm_ measured spectrophotometrically (**A**,**B**). All values were normalized to the control. Error bars indicate standard deviations from at least three independent experiments. Bars with different letters are significantly different from each other (*p* < 0.05). Representative glass slides after washing and before solubilization of the stain are shown for the 5 h (**C**) and the 2 h (**D**) treatments.

The static biofilm reduction assay as described above has been used most frequently in previous studies evaluating the efficacy of endolysins against bacterial biofilms [21,23,24,25], and it represents a valid method to assess the general capability of an enzyme to degrade biofilms. However, this model inadequately mimics situations frequently encountered in clinical and veterinarian settings as well as food processing, where biofilms are often exposed to shear forces due to the constant flow of liquids. To better account for these factors, we tested the efficacy of LysK against *S. aureus* biofilms grown under dynamic conditions, using a Biostream flow cell assembly. In a series of preliminary experiments, biofilm growth of *S. aureus* SA113 within 20 h in this setup was evaluated at various different temperatures ranging from 4 °C to 37 °C. While incubation at 4 °C resulted in no visible biofilm growth under the applied conditions, incubation at 37 °C yielded large bacterial aggregates. However, these often showed insufficient attachment to the glass surface, which resulted in frequent clogging of the flow cell and an overall low reproducibility of the experiments [45]. The temperature that yielded the best results in terms of reproducibility and, at the same time, represented a reasonable compromise between biomass production and stable attachment to the surface was 19 °C. For these reasons, this incubation temperature was chosen for all further dynamic biofilm experiments in this study. When SA113 biofilms grown under these conditions were submerged in LysK solutions of various concentrations (ranging from 0.31 to 1.25 µM) for 5 h, we observed a concentration-dependent removal of the biofilms from the glass surfaces, as demonstrated by CV staining of residual biofilms followed by spectrophotometric quantification of the solubilized stain (Figure 2A,C).

Both 1.25 and 0.63 µM LysK caused a reduction in the A_595nm_ by approximately 80% compared to the control (biofilms submerged in buffer without enzyme). At an endolysin concentration of 0.31 µM, the biofilm-disrupting effect was markedly reduced (approximately 60% reduction compared to the control). When the incubation time was shortened to 2 h, 1.25 µM LysK was still able to decrease the A_595nm_ by more than 70%, which was not significantly different (*p* > 0.05) from the 5 h treatment. Therefore, the 2 h incubation was adopted as the standard procedure in the subsequent experiments.

### 3.3. A. actinomycetemcomitans-Derived PNAG Depolymerases Disrupt S. aureus Biofilms

In an effort to identify agents with the ability to disrupt the extracellular matrix of *S. aureus* biofilms, putative PNAG depolymerases from five different *A. actinomycetemcomitans* strains (Table 1) were cloned and produced in *E. coli* as N- and C-terminally 6×His-tagged recombinant proteins. Cloning, expression, and purification by IMAC was successful for 7 out of 10 constructs, and nucleotide sequencing revealed high similarity (>90%) of all proteins with the previously described PNAG depolymerase dispersin B [27]. Cloning was not successful for the remaining three constructs.

**Figure 3:**
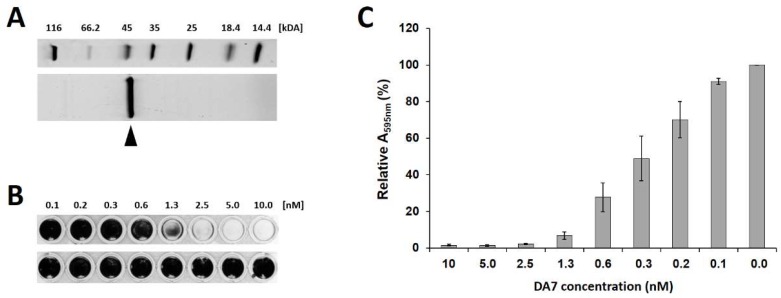
Purification and anti-biofilm activity of the polysaccharide depolymerase DA7. (**A**) Sodium dodecyl sulfate polyacrylamide gel electrophoresis (SDS-PAGE) of purified DA7 protein. The band of interest (expected molecular weight: 42.1 kDa) is marked by an arrow. (**B**) Static *S. aureus* SA113 biofilms were grown at 30 °C for 24 h, treated with increasing concentrations of DA7 (**top**) or buffer as a control (**bottom**) for 30 min at 30 °C, and stained with crystal violet (CV). (**C**) Relative A_595nm_ values measured after dissolving the CV stain on residual biofilms in 96% ethanol after DA7 treatment as shown in panel B. Values are normalized to the control, and error bars represent standard errors of the mean from three independent experiments.

All purified depolymerases were then compared for their ability to remove *S. aureus* biofilms in the static model. While all C-terminally His-tagged enzymes exhibited approximately two-fold higher activity than their N-terminally His-tagged counterparts, no significant differences in activity within the group of N-terminally His-tagged proteins or within the group of C-terminally His-tagged proteins were found [45]. Therefore, one of the C-terminally His-tagged depolymerases, DA7, which was consistently produced at high yields and purity (Figure 3A), was selected for further analysis. DA7 demonstrated high biofilm-disrupting activity, removing statically grown biofilms from polystyrene surfaces at low nanomolar concentrations (MBEC 2.5 nM; Figure 3B,C). In addition, in the dynamic model, DA7 eliminated all visible biofilm at concentrations as low as 6.25 nM (Figure S1).

### 3.4. DNase I Is Active against S. aureus SA113 Biofilms at High Concentration

Besides exopolysaccharides and proteins, extracellular DNA has been reported to be an important component of staphylococcal biofilms [46]. Aiming at a multi-enzyme approach in which various components of the extracellular matrix of *S. aureus* biofilms are targeted simultaneously, we evaluated the effect of DNase I against SA113 biofilms grown in the static model. Similar to our observations with DA7, DNAse I caused a concentration-dependent removal of the biofilms from the polystyrene surfaces (Figure S2). However, the DNAse I concentrations required to achieve effects similar to those of DA7 were relatively high, with an MBEC of 250 µg/mL (approximately 8 µM), compared to 2.5 nM for DA7. For this reason, the inclusion of DNAse I in a combination treatment with LysK was not further pursued.

### 3.5. LysK and DA7 Act Synergistically to Degrade S. aureus Biofilms in Both Static and Dynamic Models

**Figure 4:**
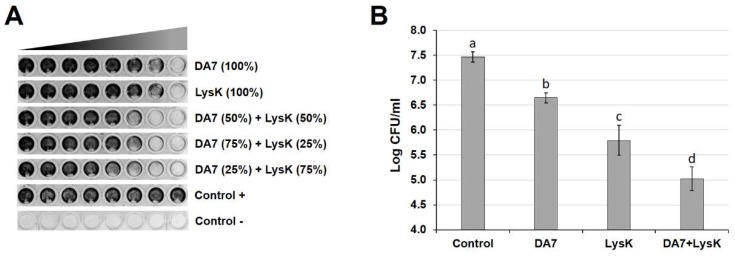
Synergistic effect of DA7 and LysK against *S. aureus* biofilms in static and dynamic models. (**A**) SA113 biofilms grown statically in 96-well plates were treated with two-fold serial dilutions of DA7, LysK, and mixtures of both enzymes at the ratios 50:50, 75:25, and 25:75, with the highest concentrations on the right and the lowest concentrations on the left of each row. After treatment, residual biofilms were stained with crystal violet. For each individual enzyme, the highest concentration used was the respective minimum biofilm eradication concentration (MBEC) and was defined as 100%. Highest concentrations of enzymes within mixtures are expressed as percentages of the respective MBECs. (**B**) Residual viable *S. aureus* on the glass surface of the Biostream flow cell after a 2 h treatment of dynamic biofilms with buffer (control), DA7 (0.625 nM), LysK (1.25 µM), or a combination of both (0.156 nM DA7 + 0.938 µM LysK, corresponding to a ratio of 25:75). Error bars represent standard deviations from at least seven independent experiments. Bars with different letters are significantly different from each other (*p* < 0.05).

The high efficacy of the depolymerase DA7 at removing *S. aureus* biofilms in both static and dynamic models led us to investigate the effect of a combined treatment with LysK and DA7 in an effort to achieve enhanced degradation of biofilm mass besides inactivation of staphylococcal cells. To this end, the efficacies of the two individual enzymes against SA113 biofilms were compared with those of enzyme mixtures at different ratios in the static biofilm model (Figure 4A). The mean ΣFBEC values obtained for the combination treatment with DA7 and LysK at the ratios 50:50, 75:25, and 25:75 in these experiments were 0.38 ± 0.14, 0.50 ± 0.00, and 0.31 ± 0.13, respectively, suggesting a synergistic effect of the two enzymes against SA113 biofilms. Based on these results, we further explored whether this synergistic effect can also be observed against biofilms grown under dynamic conditions. Therefore, SA113 biofilms grown on glass slides in four parallel Biostream flow cells were exposed to either buffer as a control, DA7, LysK, or a mixture of both enzymes at the ratio of 25:75, which had proven most effective in the static model. Residual biofilms after treatment were recovered from the glass slides and the concentrations of viable bacteria determined. All enzyme treatments significantly reduced the number of viable cells on the glass slides compared to the control treatment, with the DA7/LysK combination treatment being most effective (Figure 4B). The enzyme mixture caused a reduction in CFU/mL by approximately 2.5 log units, which was significantly (*p* < 0.05) more than the reductions caused by either individual enzyme treatment and is in agreement with the results from the static synergy assays.

**Figure 5:**
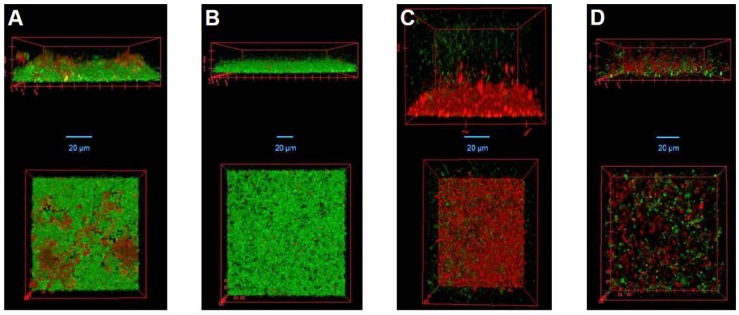
Confocal laser scanning micrographs of *S. aureus* biofilms treated with DA7, LysK, or a combination of both agents. SA113 biofilms were grown in IBIDI µ-slides for 20 h at 19 °C and then treated for 2 h with: buffer (**A**); 0.625 nM DA7 (**B**); 1.25 µM LysK (**C**); or a combination of 0.156 nM DA7 and 0.938 µM LysK (**D**). Residual biofilms after treatment were stained with LIVE/DEAD stain and then visualized by CLSM. Side views (**top**); and top views (**bottom**) of biofilm 3D reconstructions are shown. Live cells are depicted in green and dead cells as well as extracellular DNA in red.

To visualize the synergistic effect observed in both static and dynamic models, we performed CLSM on *S. aureus* biofilms grown under dynamic conditions in IBIDI µ-slides. Similar to the experiments in the Biostream flow cell, the biofilms were treated with buffer, DA7, LysK, or a mixture of both enzymes, and residual bacterial cells in the µ-slides after treatment were live/dead stained prior to microscopy. Three-dimensional reconstructions of the biofilms after various treatments generated from recorded z-stacks are shown in Figure 5. Compared to the control treatment (Figure 5A), exposure to DA7 resulted in a reduced thickness of the biofilm adhering to the slide by approximately 50%. However, the depolymerase had no visible effect on the viability of the residual cells (Figure 5B). In contrast, LysK killed the majority of the bacteria, including those close to the bottom of the biofilm, suggesting that the endolysin is able to deeply penetrate the biofilm and exert its bactericidal effect. Despite this strong killing activity, many of the dead cells (and/or extracellular DNA released upon cell lysis) appeared to remain bound within the residual biomass attached to the slides (Figure 5C). When biofilms were exposed to a mixture of DA7 and LysK at the ratio 25:75, both the overall bacterial density on the slide surface and the number of viable cells remaining after treatment were markedly reduced compared to the control, underlining the synergistic effect of the depolymerase and the endolysin against *S. aureus* biofilms.

## 4. Discussion

Bacterial biofilms are complex structures consisting of bacterial cells surrounded by a protective extracellular matrix composed of various macromolecules such as DNA, proteins, and exopolysaccharides. This network of intertwined biopolymers provides stability, contributes to cohesion of cells and adhesion to various biotic and abiotic surfaces, and limits diffusion of molecules into the biofilm [10]. This being said, it seems evident that targeting and degrading several different polymeric components of a biofilm simultaneously constitutes a promising strategy to effectively disintegrate these multi-component structures in order to eradicate unwanted bacteria. In this study, we evaluated the efficacy of the bacteriophage endolysin LysK to degrade biofilms of the important human pathogen *S. aureus*, alone and in combination with a depolymerase that digests an abundant exopolysaccharide in the biofilm matrix. It is important to note that endolysins not only kill their target cells but also further disintegrate the peptidoglycan sacculi of the dead bacteria, which by themselves constitute an important structural component of a biofilm, thereby disrupting the integrity of the entire structure.

An increasing number of studies in recent years have investigated the efficacy of various phage-derived lytic enzymes against staphylococcal biofilms [23,24,47,48,49,50,51,52,53]. The majority of these studies employed microtiter plate-based biofilm models or other static setups, and there is a general lack of endolysin efficacy studies against dynamically grown biofilms using flow cell models. One exception is a recent study by Becker et al., who reported that chimeric peptidoglycan hydrolases featuring three unique enzymatic activities were able to reduce the concentration of viable cells in *S. aureus* biofilms dynamically grown within a commercial microchannel flow cell by up to 0.89 log units within 2 h at a concentration of 1.4 µM [50]. As opposed to such microchannel systems, the Biostream flow cell used here has a volume of approximately 1 mL and allows removal of the biofilm contact surface without the necessity to break parts of the assembly. This system ensures a linear flow profile over the entire width of the chamber [31], simulating flow conditions as they are expected to occur in the bloodstream, in food processing facilities, or in milk canals within the bovine udder. When *S. aureus* biofilms grown under these conditions were treated with LysK at a concentration of 1.25 µM for 2 h, the number of residual viable cells on the slides was reduced by approximately 1.7 logs (Figure 4B). CV staining of LysK-treated biofilms revealed a noticeable difference in the way the biofilms were removed from the glass slides between the 2 h and the 5 h treatments (Figure 2). Although the overall reduction in biomass was similar for both treatment times, biofilms exposed to the endolysin for 2 h were sloughed off the surface in large fragments when applying mechanical forces during the post-treatment washes. In contrast, biofilms treated for 5 h showed a higher degree of stickiness, leaving thin layers of biomass on the slides after the washing step. This could be explained by increased release of DNA from dead bacterial cells that are exposed to the endolysin for extended periods of time. This is in agreement with the DA7-mediated biofilm removal, where no such sticky residues were observed (Figure S1). Due to its activity against the extracellular biofilm matrix but not the staphylococcal cells, no increase in extracellular DNA is to be expected in this case.

Despite our efforts to apply similar shear forces to growing biofilms in the Biostream flow cell and the IBIDI µ-slides used for CLSM, it cannot be ruled out that biofilms produced in the two systems are different, particularly regarding their 3D structure. However, the finding that the results obtained in the CLSM experiments for the different treatments are largely in agreement with those obtained with the Biostream flow cell argues against a significant impact of the flow cell system used on the susceptibility of the produced biofilms to the enzymes.

Given the high degree of similarity of DA7 and other *A. actinomycetemcomitans*-derived depolymerases investigated in this work with the previously described dispersin B [27], it is safe to assume that these proteins exhibit the same enzymatic specificity and mechanism of action in hydrolyzing poly-β(1,6)-*N*-acetyl-d-glucosamine present in the extracellular biofilm matrix of many staphylococcal strains [54]. This also explains why all depolymerases within the group of C-terminally His-tagged proteins showed similar levels of efficacy against static biofilms, and the same was true for all depolymerases within the group of N-terminally His-tagged proteins [45]. However, there was a consistent difference in activity between the C-terminally His-tagged versions of the enzymes and their N-terminally His-tagged counterparts [45]. The most likely reason for the reduced activity of the latter versions is that a His-tag fused to the N-terminus of the enzyme interferes with the conformation of the protein in an unfavorable manner or impedes access of the substrate to the catalytic center. This is in line with the finding that the enzymatically active site of dispersin B is located in the N-terminal portion of the enzyme [55].

When compared on a molar basis, the capacity of DA7 to remove *S. aureus* biomass from glass and polystyrene surfaces surpasses that of LysK by several orders of magnitude in both static and dynamic models. However, it should not be forgotten that DA7 is not bactericidal, whereas the endolysin LysK effectively kills *S. aureus* even within deeper layers of the biofilm, as demonstrated by CLSM of live/dead-stained biofilms (Figure 5). Furthermore, endolysins have been reported to be active against persister cells, a capability of high relevance in the context of biofilms, which are known to harbor a high proportion of slow- or non-growing bacteria [23]. This clearly argues for a combined application of endolysins and depolymerases, as has been investigated for LysK and DA7 in this work. The synergistic effect of these two enzymes used in combination against *S. aureus* biofilms was demonstrated in both static and dynamic models. It can most likely be attributed to facilitated access for LysK to *S. aureus* cells embedded within the biofilm matrix when PNAG, an important component of this matrix, is degraded by DA7. Likewise, disintegration of bacterial cell walls by LysK may enable DA7 to penetrate into deeper layers of the biofilm, which overall leads to a more effective destabilization of the three-dimensional structure. Besides the observed synergy, the activities of the individual enzymes were found to be similar when comparing static and dynamic models. In both cases, LysK and DA7 removed the majority of visible biofilm at low micromolar and low nanomolar concentrations, respectively, suggesting that dynamically grown biofilms do not show a substantially higher level of recalcitrance against the enzymes under the conditions applied here. While different surface materials may affect the adhesion capacity of bacterial cells, it has been suggested that polystyrene (as used in our static model) and glass (as used in the Biostream flow cell) do not significantly differ in the attachment capacity of various organisms, including *S. aureus* [56]. However, it should be noted that direct comparison of results from the static and dynamic models is difficult due to the different growth and treatment conditions applied in the two models.

Staining of biofilms with CV as it has been applied here in both static and dynamic models is one of the most widely used methods for quantification of adhered biomass. It offers several advantages such as versatility and high throughput capability. Furthermore, it avoids the necessity to detach biofilms from their growth surfaces, as required for enumeration of viable cells. At the same time, one needs to be aware of the drawbacks of this method. These include a lack of reproducibility and sensitivity and a possible over- or underestimation of biofilm mass in dependence of the washing steps (reviewed in [57]).

*S. aureus* strain SA113, which was used in most static and dynamic biofilm experiments in this study, is a reportedly strong biofilm former that is known to produce PNAG as a major component of its extracellular matrix [43]. This explains the high efficacy of the PNAG-depolymerase DA7 against SA113 biofilms, as shown here. At the same time, it implies that DA7 is inactive against biofilms of *S. aureus* strains incapable of PNAG production or for which PNAG is not a major extracellular matrix component, as has been demonstrated for the DA7-homologue dispersin B [58]. It is important to note that the *icaADBC* locus, which is responsible for PNAG production, is present in the majority of all *S. aureus* clinical isolates [59], and that production of PNAG has been shown to be a crucial factor for *S. aureus* pathogenesis in murine models of systemic infection [60] and in *S. aureus*-induced bovine mastitis [14]. One non-dispersin B-like exopolysaccharide depolymerase named Dpo7 with activity against *S. aureus* biofilms has recently been described [29]. However, the exact molecular target of this phage-derived enzyme is still unknown.

Aiming at a multi-enzyme approach to eradicate *S. aureus* biofilms, we also investigated the ability of DNase I to reduce SA113 biofilms by degrading extracellular DNA. Our results are in agreement with previous studies, which had found that DNase I compromised the integrity of biofilms formed by various *S. aureus* strains, leading to their detachment [26,58,61]. However, under the conditions applied in our work, the DNase I concentrations required to achieve similar effects as for DA7 were more than 1000-fold higher when compared on a molar basis. Therefore, DNase I was not included in the synergy experiments in this study. However, similar to what has been discussed for DA7, susceptibility to DNase I can vary from strain to strain, likely depending on the proportion of extracellular DNA within the biofilm matrix.

Strain-dependent differences in efficacy against *S. aureus* biofilms were also observed for LysK, even though the endolysin displayed strong activity against the majority of the strains tested here in the static model. This is consistent with a previous report, in which the staphylococcal phage endolysin LysGH15 was shown to degrade biofilms of more than 30 staphylococcal strains from different species (*S. aureus*, *S. epidermidis*, *S. hominis*, and *S. haemolyticus*) grown for 24 or 72 h in a static model [47]. LysGH15 is almost identical with LysK, differing from the latter in only four amino acids [19]. Furthermore, LysK has demonstrated strong lytic activity against planktonic cells of a large set of staphylococcal strains, including clinical *S. aureus* isolates (MSSA and MRSA), bovine mastitis isolates, coagulase-negative staphylococci, and multiple mutant strains with altered surface structures [21]. Taken together, these results provide further evidence that LysK is a potent staphylolytic enzyme with a broad spectrum of activity against planktonic cells and biofilms. However, further studies are required to investigate whether this holds true when LysK is used against dynamically grown biofilms of multiple strains, alone or in combination with DA7.

It is important to understand that the growth conditions (and growth temperatures in particular) applied in the biofilm experiments in this work were not chosen in order to optimally simulate conditions found in either clinical/veterinarian or food safety environments or to allow direct comparison between the static and dynamic models, but in an effort to produce the most stable and reproducible biofilms within each given setup. When aiming at specific applications in either of these settings, further experiments beyond the scope of the current study are advisable. These could include investigating the impact of various parameters such as media composition, growth temperature, incubation time, surface material and surface coatings on the growth of *S. aureus* biofilms and their susceptibility to LysK and DA7. Further, it could be relevant to investigate mixed-species biofilms and their susceptibility to these combined treatment strategies, since they are prevalent in most settings outside the laboratory [62,63]. Moreover, it could be interesting, at least from a scientific perspective, to assess the potential of both enzymes to prevent biofilm growth as opposed to disruption of mature biofilms. While preventive application may not be viable in food production due to the high costs associated with it, it may be feasible in certain niche medical applications, e.g., following surgery for insertion of prosthetic joints or catheters.

In the context of medical and veterinarian applications, a combination of our enzyme-based approach with classical antibiotic treatment, as has been suggested previously [64], could constitute an effective therapy against *S. aureus* infections involving biofilms. In bovine mastitis treatment, biofilm-degrading enzymes could contribute to the declogging of milk ducts congested by aggregates of bacterial and somatic cells in order to facilitate penetration of the udder by antibacterial agents administered intramammarily. Finally, biofilm-disrupting enzymes could be part of an effective hurdle technology applied in food production and processing facilities to alleviate the risk of biofilm-mediated contamination of food products.

## Figures and Tables

**Table 1:** Bacterial strains used in this work.

Strain	Characteristics	Source, Reference
*E. coli* XL1-Blue MRF’	Cloning and expression strain	^1^
*E. coli* BL21-Gold(DE3)	Cloning and expression strain	^1^
*S. aureus* SA113 (ATCC 35556)	Strong biofilm former	^2^, [32]
*S. aureus* RN6911	*agr*-deficient mutant strain	^3^, [33]
*S. aureus* SA001	Bovine mastitis isolate	^4^, [34]
*S. aureus* Mastidis	Bovine mastitis isolate	^5^
*S. aureus* 305 (Newbould) (ATCC 29740)	Bovine mastitis isolate	^6^, [35]
*S. aureus* R174	Food isolate	^5^
*S. aureus* R177	Food isolate	^5^
*S. aureus* R191	Food isolate	^5^
*S. aureus* R192	Food isolate	^5^
*S. aureus* 319	Food isolate	^5^
*S. aureus* 350	Food isolate	^5^
*S. aureus* 2971	Food isolate	^5^
*S. aureus* 95	Food isolate	^5^
*A. actinomycetemcomitans* OMZ 542	Source of depolymerase	^7^
*A. actinomycetemcomitans* OMZ 247	Source of depolymerase	^7^
*A. actinomycetemcomitans* OMZ 295	Source of depolymerase	^7^
*A. actinomycetemcomitans* OMZ 296	Source of depolymerase	^7^
*A. actinomycetemcomitans* OMZ 534	Source of depolymerase	^7^

^1^ Agilent Technologies, Santa Clara, CA, USA; ^2^ Andreas Peschel, University of Tübingen, Tübingen, Germany; ^3^ Brigitte Berger-Bächi, University of Zurich, Zurich, Switzerland; ^4^ Yasunori Tanji, Tokyo Institute of Technology, Yokohama, Japan; ^5^ Roger Stephan, University of Zurich, Zurich, Switzerland; ^6^ David M. Donovan, ARS, USDA, Beltsville, MD, USA; ^7^ Georgios Belibasakis, Karolinska Institutet, Stockholm, Sweden (formerly at University of Zurich, Zurich, Switzerland).

## Supplementary Materials

The following are available online at http://www.mdpi.com/1999-4915/10/8/438/s1: Figure S1: Efficacy of DA7 against dynamic *S. aureus* SA113 biofilms grown on glass surfaces in the Biostream flow cell. Figure S2: Activity of DNAse I against *S. aureus* SA113 biofilms grown in a 96-well plate under static conditions.

## References

[1] Götz F., Bannerman T., Schleifer K.H., Falkow S., Rosenberg E., Schleifer K.H., Stackebrandt E. (2006). The Genera *Staphylococcus* and *Macrococcus*. The Prokaryotes.

[2] Lowy F.D. (1998). *Staphylococcus* aureus infections. N. Engl. J. Med..

[3] Thurnheer T., Belibasakis G.N. (2015). Integration of non-oral bacteria into in vitro oral biofilms. Virulence.

[4] Thurnheer T., Belibasakis G.N. (2016). Incorporation of staphylococci into titanium-grown biofilms: An in vitro “submucosal” biofilm model for peri-implantitis. Clin. Oral Implants Res..

[5] Charalampakis G., Belibasakis G.N. (2015). Microbiome of peri-implant infections: Lessons from conventional, molecular and metagenomic analyses. Virulence.

[6] Fisher E.L., Otto M., Cheung G.Y.C. (2018). Basis of Virulence in Enterotoxin-Mediated Staphylococcal Food Poisoning. Front. Microbiol..

[7] Sordillo L.M., Streicher K.L. (2002). Mammary gland immunity and mastitis susceptibility. J. Mammary Gland Biol. Neoplasia.

[8] Ben-David D., Novikov I., Mermel L.A. (2009). Are there differences in hospital cost between patients with nosocomial methicillin-resistant *Staphylococcus aureus* bloodstream infection and those with methicillin-susceptible *S. aureus* bloodstream infection?. Infect. Control Hosp. Epidemiol..

[9] Götz F. (2002). *Staphylococcus* and biofilms. Mol. Microbiol..

[10] Abee T., Kovacs A.T., Kuipers O.P., van der Veen S. (2011). Biofilm formation and dispersal in Gram-positive bacteria. Curr. Opin. Biotechnol..

[11] Costerton J.W., Lewandowski Z., Caldwell D.E., Korber D.R., Lappin-Scott H.M. (1995). Microbial biofilms. Annu. Rev. Microbiol..

[12] Otto M. (2008). Staphylococcal biofilms. Curr. Top. Microbiol. Immunol..

[13] Brooks J.D., Flint S.H. (2008). Biofilms in the food industry: Problems and potential solutions. Int. J. Food Sci. Technol..

[14] Gomes F., Saavedra M.J., Henriques M. (2016). Bovine mastitis disease/pathogenicity: Evidence of the potential role of microbial biofilms. Pathog. Dis..

[15] Young R. (1992). Bacteriophage lysis: Mechanism and regulation. Microbiol. Rev..

[16] Loessner M.J. (2005). Bacteriophage endolysins—Current state of research and applications. Curr. Opin. Microbiol..

[17] Fischetti V.A. (2005). Bacteriophage lytic enzymes: Novel anti-infectives. Trends Microbiol..

[18] Schmelcher M., Donovan D.M., Loessner M.J. (2012). Bacteriophage endolysins as novel antimicrobials. Future Microbiol..

[19] Haddad Kashani H., Schmelcher M., Sabzalipoor H., Seyed Hosseini E., Moniri R. (2018). Recombinant Endolysins as Potential Therapeutics against Antibiotic-Resistant *Staphylococcus aureus*: Current Status of Research and Novel Delivery Strategies. Clin. Microbiol. Rev..

[20] Nelson D.C., Schmelcher M., Rodriguez-Rubio L., Klumpp J., Pritchard D.G., Dong S., Donovan D.M. (2012). Endolysins as antimicrobials. Adv. Virus Res..

[21] Schmelcher M., Shen Y., Nelson D.C., Eugster M.R., Eichenseher F., Hanke D.C., Loessner M.J., Dong S., Pritchard D.G., Lee J.C. (2015). Evolutionarily distinct bacteriophage endolysins featuring conserved peptidoglycan cleavage sites protect mice from MRSA infection. J. Antimicrob. Chemother..

[22] O’Flaherty S., Coffey A., Meaney W., Fitzgerald G.F., Ross R.P. (2005). The recombinant phage lysin LysK has a broad spectrum of lytic activity against clinically relevant staphylococci, including methicillin-resistant *Staphylococcus aureus*. J. Bacteriol..

[23] Gutierrez D., Ruas-Madiedo P., Martinez B., Rodriguez A., Garcia P. (2014). Effective removal of staphylococcal biofilms by the endolysin LysH5. PLoS ONE.

[24] Jun S.Y., Jung G.M., Yoon S.J., Oh M.D., Choi Y.J., Lee W.J., Kong J.C., Seol J.G., Kang S.H. (2013). Antibacterial properties of a pre-formulated recombinant phage endolysin, SAL-1. Int. J. Antimicrob. Agents.

[25] Sass P., Bierbaum G. (2007). Lytic activity of recombinant bacteriophage phi11 and phi12 endolysins on whole cells and biofilms of *Staphylococcus aureus*. Appl. Environ. Microbiol..

[26] Kaplan J.B., LoVetri K., Cardona S.T., Madhyastha S., Sadovskaya I., Jabbouri S., Izano E.A. (2012). Recombinant human DNase I decreases biofilm and increases antimicrobial susceptibility in staphylococci. J. Antibiot..

[27] Kaplan J.B., Ragunath C., Ramasubbu N., Fine D.H. (2003). Detachment of *Actinobacillus actinomycetemcomitans* biofilm cells by an endogenous beta-hexosaminidase activity. J. Bacteriol..

[28] Kaplan J.B., Ragunath C., Velliyagounder K., Fine D.H., Ramasubbu N. (2004). Enzymatic detachment of *Staphylococcus epidermidis* biofilms. Antimicrob. Agents Chemother..

[29] Gutierrez D., Briers Y., Rodriguez-Rubio L., Martinez B., Rodriguez A., Lavigne R., Garcia P. (2015). Role of the Pre-neck Appendage Protein (Dpo7) from Phage vB_SepiS-phiIPLA7 as an Anti-biofilm Agent in Staphylococcal Species. Front. Microbiol..

[30] Schmelcher M., Loessner M.J. (2016). Bacteriophage endolysins: Applications for food safety. Curr. Opin. Biotechnol..

[31] Vanzieleghem T., Mahillon J., Jeanmart H., Degand S., Dupont C., Ladeuze S. (2014). Fluidic Device for Studying of Surface-Dwelling Multicellular Layers and Microbial Biofilms. Eur. Pat..

[32] Iordanescu S., Surdeanu M. (1976). Two restriction and modification systems in *Staphylococcus aureus* NCTC8325. J. Gen. Microbiol..

[33] Novick R.P., Ross H.F., Projan S.J., Kornblum J., Kreiswirth B., Moghazeh S. (1993). Synthesis of staphylococcal virulence factors is controlled by a regulatory RNA molecule. EMBO J..

[34] Synnott A.J., Kuang Y., Kurimoto M., Yamamichi K., Iwano H., Tanji Y. (2009). Isolation from sewage influent and characterization of novel *Staphylococcus aureus* bacteriophages with wide host ranges and potent lytic capabilities. Appl. Environ. Microbiol..

[35] Prasad L.B., Newbould F.H. (1968). Inoculation of the bovine teat duct with *Staph. aureus*: The relationship of teat duct length, milk yield and milking rate to development of intramammary infection. Can. Vet. J..

[36] Sambrook J., Fritsch E.F., Maniatis T. (1989). Molecular Cloning: A Laboratory Manual.

[37] Verbree C.T., Dätwyler S.M., Meile S., Eichenseher F., Donovan D.M., Loessner M.J., Schmelcher M. (2018). Corrected and Republished from: Identification of Peptidoglycan Hydrolase Constructs with Synergistic Staphylolytic Activity in Cow’s Milk. Appl. Environ. Microbiol..

[38] Schmelcher M., Loessner M.J. (2014). Use of bacteriophage cell wall-binding proteins for rapid diagnostics of *Listeria*. Methods Mol. Biol..

[39] Vanzieleghem T., Couniot N., Herman-Bausier P., Flandre D., Dufrene Y.F., Mahillon J. (2016). Role of Ionic Strength in Staphylococcal Cell Aggregation. Langmuir.

[40] Jones R.N., Barry A.L., Gavan T.L., Washington Ii J.A., Balows A., Hausler J.W.J., Shadomy H.J. (1985). Susceptibility tests: Microdilution and macrodilution broth procedures. Manual of Clinical Microbiology.

[41] Hall M.J., Middleton R.F., Westmacott D. (1983). The fractional inhibitory concentration (FIC) index as a measure of synergy. J. Antimicrob. Chemother..

[42] Abramoff M.D., Magalhaes P.J., Ram S.J. (2004). Image processing with Image. Biophotonics Int..

[43] Cramton S.E., Gerke C., Schnell N.F., Nichols W.W., Götz F. (1999). The intercellular adhesion (*ica*) locus is present in *Staphylococcus aureus* and is required for biofilm formation. Infect. Immun..

[44] Boles B.R., Horswill A.R. (2008). Agr-mediated dispersal of *Staphylococcus aureus* biofilms. PLoS Pathog..

[45] Schmelcher M. (2014). Removal of *Staphylococcus aureus* Biofilms from Abiotic Surfaces.

[46] Montanaro L., Poggi A., Visai L., Ravaioli S., Campoccia D., Speziale P., Arciola C.R. (2011). Extracellular DNA in biofilms. Int. J. Artif. Organs.

[47] Zhang Y., Cheng M., Zhang H., Dai J., Guo Z., Li X., Ji Y., Cai R., Xi H., Wang X. (2018). Antibacterial effects of phage lysin LysGH15 on planktonic cells and biofilms of diverse staphylococci. Appl. Environ. Microbiol..

[48] Melo L.D.R., Brandao A., Akturk E., Santos S.B., Azeredo J. (2018). Characterization of a New *Staphylococcus aureus* Kayvirus Harboring a Lysin Active against Biofilms. Viruses.

[49] Schuch R., Khan B.K., Raz A., Rotolo J.A., Wittekind M. (2017). Bacteriophage Lysin CF-301, a Potent Antistaphylococcal Biofilm Agent. Antimicrob. Agents Chemother..

[50] Becker S.C., Roach D.R., Chauhan V.S., Shen Y., Foster-Frey J., Powell A.M., Bauchan G., Lease R.A., Mohammadi H., Harty W.J. (2016). Triple-acting Lytic Enzyme Treatment of Drug-Resistant and Intracellular *Staphylococcus aureus*. Sci. Rep..

[51] Drilling A.J., Cooksley C., Chan C., Wormald P.J., Vreugde S. (2016). Fighting sinus-derived *Staphylococcus aureus* biofilms in vitro with a bacteriophage-derived muralytic enzyme. Int. Forum Allergy Rhinol..

[52] Chopra S., Harjai K., Chhibber S. (2015). Potential of sequential treatment with minocycline and *S. aureus* specific phage lysin in eradication of MRSA biofilms: An in vitro study. Appl. Microbiol. Biotechnol..

[53] Fenton M., Keary R., McAuliffe O., Ross R.P., O’Mahony J., Coffey A. (2013). Bacteriophage-Derived Peptidase CHAP(K) Eliminates and Prevents Staphylococcal Biofilms. Int. J. Microbiol..

[54] Fazekas E., Kandra L., Gyemant G. (2012). Model for β-1,6-*N*-acetylglucosamine oligomer hydrolysis catalysed by DispersinB, a biofilm degrading enzyme. Carbohydr. Res..

[55] Ramasubbu N., Thomas L.M., Ragunath C., Kaplan J.B. (2005). Structural analysis of dispersin B, a biofilm-releasing glycoside hydrolase from the periodontopathogen *Actinobacillus actinomycetemcomitans*. J. Mol. Biol..

[56] Mafu A.A., Plumety C., Deschenes L., Goulet J. (2011). Adhesion of Pathogenic Bacteria to Food Contact Surfaces: Influence of pH of Culture. Int. J. Microbiol..

[57] Azeredo J., Azevedo N.F., Briandet R., Cerca N., Coenye T., Costa A.R., Desvaux M., Di Bonaventura G., Hébraud M., Jaglic Z. (2017). Critical review on biofilm methods. Crit. Rev. Microbiol..

[58] Izano E.A., Amarante M.A., Kher W.B., Kaplan J.B. (2008). Differential roles of poly-*N*-acetylglucosamine surface polysaccharide and extracellular DNA in *Staphylococcus aureus* and *Staphylococcus epidermidis* biofilms. Appl. Environ. Microbiol..

[59] O’Gara J.P. (2007). *ica* and beyond: Biofilm mechanisms and regulation in *Staphylococcus epidermidis* and *Staphylococcus aureus*. FEMS Microbiol. Lett..

[60] Kropec A., Maira-Litran T., Jefferson K.K., Grout M., Cramton S.E., Götz F., Goldmann D.A., Pier G.B. (2005). Poly-*N*-acetylglucosamine production in *Staphylococcus aureus* is essential for virulence in murine models of systemic infection. Infect. Immun..

[61] Waryah C.B., Wells K., Ulluwishewa D., Chen-Tan N., Gogoi-Tiwari J., Ravensdale J., Costantino P., Gokcen A., Vilcinskas A., Wiesner J. (2017). In Vitro Antimicrobial Efficacy of Tobramycin Against *Staphylococcus aureus* Biofilms in Combination With or Without DNase I and/or Dispersin B: A Preliminary Investigation. Microb. Drug Resist..

[62] Elias S., Banin E. (2012). Multi-species biofilms: Living with friendly neighbors. FEMS Microbiol. Rev..

[63] Burmølle M., Ren D., Bjarnsholt T., Sørensen S.J. (2014). Interactions in multispecies biofilms: Do they actually matter?. Trends Microbiol..

[64] Djurkovic S., Loeffler J.M., Fischetti V.A. (2005). Synergistic killing of *Streptococcus pneumoniae* with the bacteriophage lytic enzyme Cpl-1 and penicillin or gentamicin depends on the level of penicillin resistance. Antimicrob. Agents Chemother..

