# Supplementary material for: Synergistic Removal of Static and Dynamic Staphylococcus aureus Biofilms by Combined Treatment with a Bacteriophage Endolysin and a Polysaccharide Depolymerase
Source: Viruses. 2018 Aug 18;10(8):438. doi: 10.3390/v10080438 (PMC6116285; doi:10.3390/v10080438)
Supplement: Supplementary file 1 [file viruses-10-00438-s001.pdf]

Supplemental Material for "Synergistic removal of static and dynamic *Staphylococcus aureus* biofilms by combination treatment with a bacteriophage endolysin and a polysaccharide depolymerase"

Supplemental Figure 1.

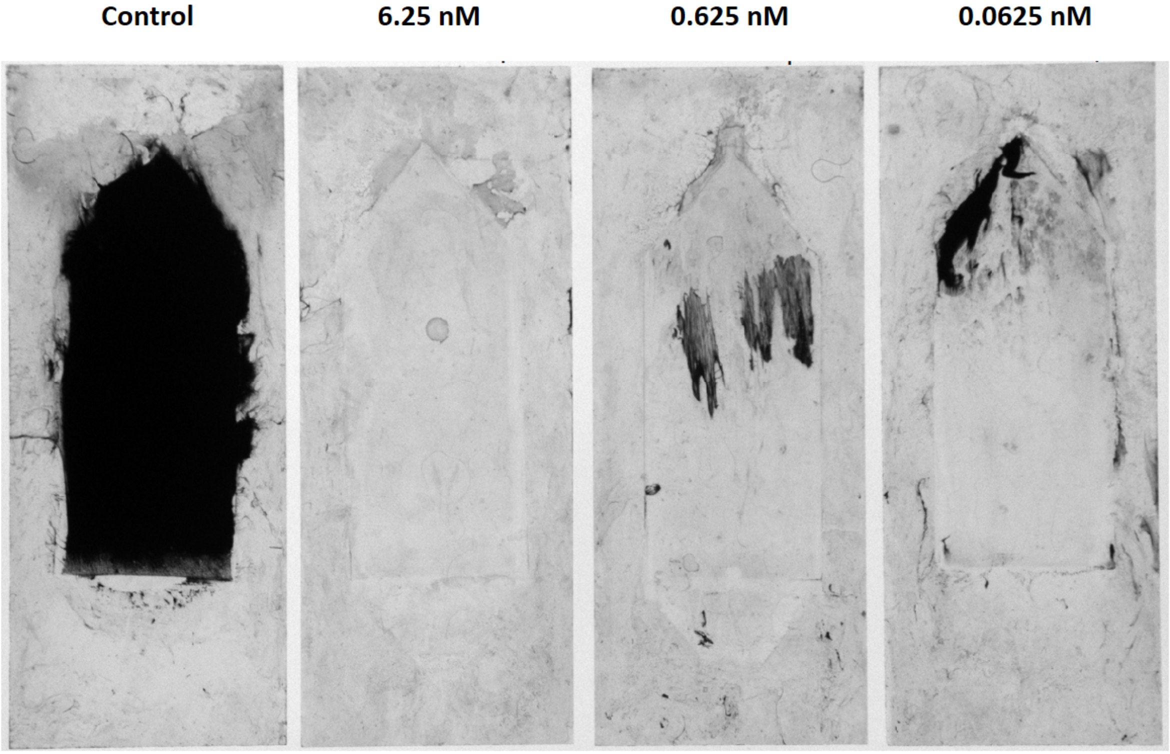

Figure S1. Efficacy of DA7 against dynamic *S. aureus* SA113 biofilms grown on glass surfaces in the Biostream flow cell. Biofilms were grown for 20 h at 19°C under continuous flow of medium. Glass slides were then submerged in solutions of DA7 at different concentrations or buffer as a control for 2 h and stained with crystal violet.

Supplemental Figure 2

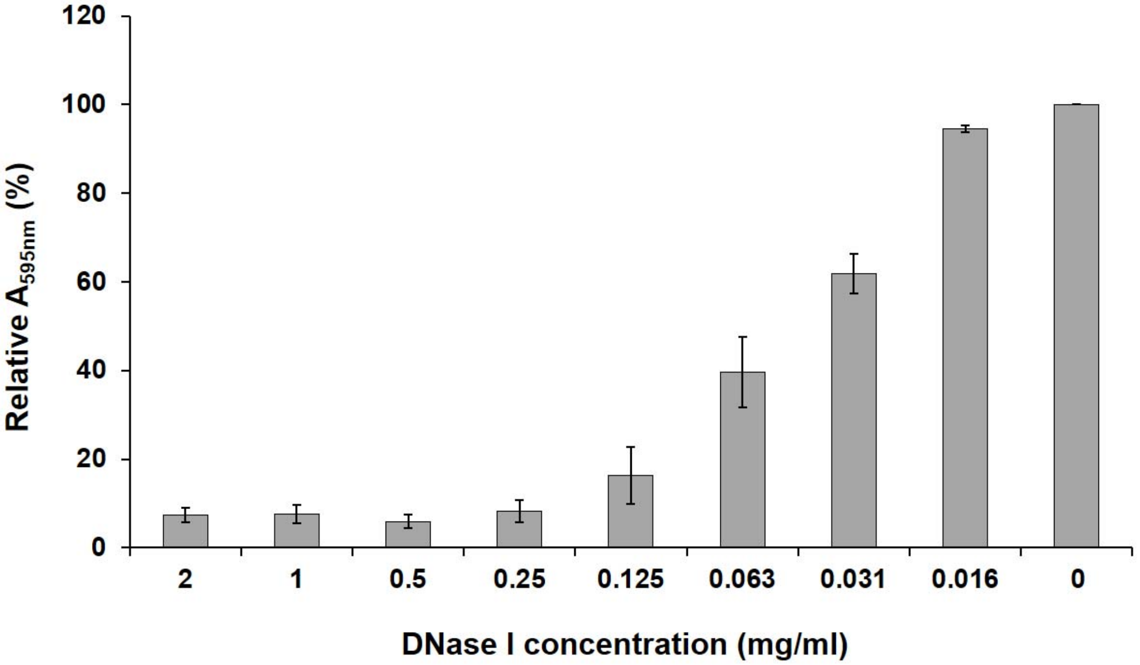

Figure S2. Activity of DNAse I against *S. aureus* SA113 biofilms grown in a 96-well plate under static conditions. Biofilms were treated with DNAse I at different concentrations or buffer as a control (0) for 1 h, and residual biofilms after treatment were stained with crystal violet (CV). After dissolving the CV in 96% ethanol, the A_{595} of each well was measured spectrophotometrically. All values were normalized to the control. Error bars indicate standard deviations from three independent experiments.
